# Brain Networks, Neurotransmitters and Psychedelics: Towards a Neurochemistry of Self-Awareness

**DOI:** 10.1007/s11910-024-01353-y

**Published:** 2024-07-09

**Authors:** Daniel C. Mograbi, Rafael Rodrigues, Bheatrix Bienemann, Jonathan Huntley

**Affiliations:** 1 Department of Psychology, Pontifical Catholic University of Rio de Janeiro, Rio de Janeiro, Brazil; 2 Institute of Psychiatry, Psychology & Neuroscience, King’s College London, London, UK; 3 Division of Psychiatry, University College London, London, UK; 4 Wellcome Centre for Human Neuroimaging, University College London, London, UK

**Keywords:** Interoception, agency, metacognition, emotional regulation, psilocybin, LSD

## Abstract

**Purpose of Review:**

Self-awareness can be defined as the capacity of becoming the object of one’s own awareness and, increasingly, it has been the target of scientific inquiry. Self-awareness has important clinical implications, and a better understanding of the neurochemical basis of self-awareness may help clarifying causes and developing interventions for different psychopathological conditions. The current article explores the relationship between neurochemistry and self-awareness, with special attention to the effects of psychedelics.

**Recent Findings:**

The functioning of self-related networks, such as the default-mode network and the salience network, and how these are influenced by different neurotransmitters is discussed. The impact of psychedelics on self-awareness is reviewed in relation to specific processes, such as interoception, body ownership, agency, metacognition, emotional regulation and autobiographical memory, within a framework based on predictive coding.

**Summary:**

Improved outcomes in emotional regulation and autobiographical memory have been observed in association with the use of psychedelics, suggesting higher-order self-awareness changes, which can be modulated by relaxation of priors and improved coping mechanisms linked to cognitive flexibility. Alterations in bodily self-awareness are less consistent, being potentially impacted by doses employed, differences in acute/long-term effects and the presence of clinical conditions. Future studies investigating the effects of different molecules in rebalancing connectivity between resting-state networks may lead to novel therapeutic approaches and the refinement of existing treatments.

## Introduction

Self-awareness has been a perennial question for our species. Different religious and philosophical traditions have tackled this subject, but only more recently has it been the target of scientific inquiry. Self-awareness can be defined as the capacity to take oneself as the object of awareness [1]. This definition, however, does not imply that the self and, consequently, self-awareness are unitary concepts. Rather, self-related information from different sources enters the focus of awareness at any given time, leading to a variety of self-awareness processes [2], which, when integrated, result in an emergent sense of self-awareness [3]. In this perspective, self-awareness operates similarly to consciousness in general, emerging from the integration of information from multiple implicit and explicit sources.

The past 20 years have witnessed a systematic attempt to develop a cognitive neuroscience of self-awareness [4]. Studies measuring brain activity in healthy individuals, as well as investigations involving people with neurological and psychiatric disorders, have revealed neural networks associated with processes such as interoception [5], body ownership [6], and metacognition [7]. Nevertheless, progress has been relatively slower in relation to the identification of neurochemical bases of self-awareness. This is particularly important in terms of providing mechanistic explanations. Although identifying brain regions is a preliminary step to the cognitive neuroscience of self-awareness, the mechanisms driving this process will never be fully understood without an appreciation of the dynamics and the effects of neurotransmitters on neural networks. Additionally, a finer understanding of the neurochemical basis of self-awareness may help to elucidate the causes of different psychopathological conditions, fostering the development and refinement of pharmacological interventions for these disorders.

Psychedelics represent a class of substances that not only have considerable therapeutic potential, but that have also been consistently associated, in user reports, with alterations in self-awareness. Psychedelics cause profound effects on psychological functioning, being characterized by altered states of consciousness marked by changes in perception, emotion, and cognition [8, 9]. Classic psychedelics, such as ayahuasca, lysergic acid diethylamide (LSD), mescaline and psilocybin, act mainly through agonism of serotonin 2A receptors [10], but also interact with other neurotransmitters. Substances such as 3,4-methyl​enedioxy​methamphetamine (MDMA), ketamine and salvia exhibit a different profile in terms of binding affinities to different receptor types, but given their overlap with classic psychedelics in terms of subjective experiences, these are sometimes grouped together. Although psychedelics accessible in nature, such as ayahuasca, mescaline, and psilocybin, have been used for centuries by traditional indigenous cultures, scientific exploration into the therapeutic effects of these substances gained traction from the 1950s [11], after the synthesis of LSD. The past years have witnessed an acceleration in the research into the therapeutic potential of psychedelics, with relevant preliminary results for conditions such as depression (ayahuasca [12, 13], psilocybin [14, 15]), anxiety (LSD [16]), and trauma (MDMA [17, 18]). The use of psychedelics in conjunction with psychotherapy support has been suggested as a potential paradigm shift in psychiatry [19].

Some of the earliest scientific descriptions of the effects of psychedelics emphasized changes in selfhood, for example with anomalous bodily experiences and a sense of unity with the environment after consumption of mescaline [20, 21]. Similarly, Huxley described mescaline as leading to a state of “egolessness” and a feeling of oneness with everything [22]. More recent research has reaffirmed these changes in self-awareness, with blurring of boundaries between self and the world, and feelings of unity with the universe (“oceanic feeling”), as a central axis of the psychedelic experience, across different substances [8, 23]. This has been generally referred to as “ego dissolution”, and although the phenomenon has been described also in relation to negative experiences [24], some studies have suggested that these changes in sense of self and self-awareness may be a predictor of positive outcomes in conditions treated by psychedelic substances [23, 25, 26].

Considering the above, the purpose of this review is to explore the neurochemical basis of self-awareness and discuss its potential modulation by psychedelics. We first review the neural correlates of self-related networks and current evidence on how these may be influenced by the action of different neurotransmitters. Predictive coding is then discussed as a framework to understand the impact of psychedelics, including on self-awareness. This is followed by a review of the action of psychedelics on specific self-awareness processes. Finally, future research directions to test existing frameworks are explored.

## Self-Related Networks and their Neurochemistry

### Self-Related Brain Networks

Although it is not possible to reduce the self to any specific brain region, with some suggesting this could amount to a category error [27]*,* efforts have been made to explore the neural correlates of self-related processes. Earlier attempts suggested a variety of networks linked to self-processes, already highlighting the role of cortical midline structures in self-representation, monitoring, and integration [28, 29]. Increasingly, in the past decades, neuroimaging techniques have revealed aspects of the self that are associated with the dynamic and coordinated activity of a large-scale brain network. This network has been referred to as the Default Mode Network (DMN).

The notion that the brain is active even during rest and passive conditions had already been suggested in the first half of the 20^th^ century, with neuroimaging techniques in the 1990s providing consistent support for this idea (for a detailed history of research into the DMN, see [30] and [31]). The DMN is usually more activated in human subjects during mind-wandering activity [32] or resting state [33], and less activated during cognitively demanding, externally focused tasks [33, 34]. Studies that involve tasks related to self traits and/or self vs. other distinction were among the first to demonstrate that DMN nodes could be largely involved in self-perception and -differentiation. Studies comparing self-referential and resting-state processes have identified overlapping activation in the mPFC (medial prefrontal cortex), PCC (posterior cingulate cortex), and left AG (angular gyrus) [35, 36]. Notably, it was also discovered that these three areas exhibit enhanced activation during self-referential processing compared with resting baseline activity [35]. These studies suggest that the baseline neural activity of humans is highly associated with self-referential processing. DMN activity and connectivity have also been correlated with subjective awareness in people with disorders of consciousness, such as vegetative or minimally conscious states. For example, results from Vanhaudenhuyse et al [37] indicate that DMN connectivity is decreased in severely brain-damaged patients, proportionally to the degree of consciousness impairment. Similarly, Hannawi et al. [38] suggest markedly reduced activity within anatomical structures that have been linked to DMN in people with disorders of consciousness.

The distinct roles of the DMN nodes in self-referential processing are not yet fully understood, but the mPFC appears to be particularly sensitive to differentiating self from others [35, 39]. Additionally, the mPFC seems to be more specifically engaged by demands for cognitive elaboration, evaluation, and emotion reappraisal [40], while the left AG seems to be more involved in the retrieval of semantic and personally relevant information [41, 42]. The PCC seems to upregulate the activity of other DMN nodes during self-related mental processes [35], and has also been shown to be linked to memory aspects of self-awareness, including autobiographical memory recall [43].

The contribution of other networks for self-related processing has also been discussed. For example, it has been suggested that the salience network (SN) may be responsible for embodied aspects of the self [44]. The general role of the SN would be to identify relevant internal and external stimuli [45], as well as being responsible for switching activation/deactivation of other networks, such as the DMN and central executive network [46]. Its main functional hubs are the dorsal anterior cingulate cortex and the insula [47]. The latter is particularly important for multisensory integration and interoceptive processing [5, 48]. Alterations in this network have been observed across disorders, for example in Alzheimer’s disease [49] and schizophrenia [50], in which self-alterations are prominent [51, 52]*.*

### The Role of Dopamine and GABA on DMN Activity

Despite these advances in the investigation of the DMN, Alves et al. [53] argue that most research has focused on the cortex, with less attention given to subcortical structures. They propose a neuroanatomical model of the DMN that also includes subcortical structures such as the basal forebrain, cholinergic nuclei, anterior and mediodorsal thalamic nuclei [53]. In particular, the basal forebrain comprises a group of neurochemically diverse nuclei, involved in dopaminergic, cholinergic, and serotonergic pathways, that are crucial in the pathophysiology of the diseases that affect DMN connectivity. Alves et al. [53] also suggest an influence of the mesolimbic dopaminergic pathway in several brain networks associated with the DMN, arguing that the DMN might have a putative role in the integration of cholinergic and dopaminergic systems dedicated to memory and emotion. This highlights that neurochemical influences in self-awareness should be considered as a dynamic balance between multiple neurochemical networks and not a specific one.

The DMN has been found to exhibit a bidirectional interaction, with oscillatory activity synchronized throughout a range of beta and gamma frequencies (25–100Hz), with maximum synchronization at 40Hz in the gamma range [54]. There is accumulating evidence that the functional magnetic resonance imaging (fMRI) hemodynamic response signal attributed to DMN and self-referential processing is correlated with neuronal activity in the gamma band (EEG studies [55, 56]), and in particular high-gamma band (intracranial EEG studies [57–60]. Synchronized oscillations in the gamma range between medial prefrontal and parietal regions are linked to cognitive function [61, 62], with causality potentially being inferred from the electrophysiological findings of Lou et al. [63] and Luber et al. [64]. This synchronization is known to facilitate self-awareness [65] and its reduction is associated with addiction to gambling and related problems, in which impaired self-awareness is a feature [66].

In humans, Hall et al. [67] showed that GABA-ergic stimulation induced gamma power increase in the prefrontal cortex. The GABA receptors are constructed as ligand ion channels, constituting a pore between five protein complexes, regulating the passing of negative chloride ions, and the generation of electrical pulses when stabilized in an open conformation [68]. Stephens et al. [68] argue that this stabilization is achieved by binding GABA to the pore complex; the binding, however, is not specific to GABA. The affinity of other molecules, endogenous (e.g. dopamine) or not (e.g. muscimol, present in Amanita muscaria [69]), depends on the protein composition of the molecules constituting the pore, with potential abnormalities in the subtype composition of the pore in disorders such as addiction [68].

Some authors suggest that self-awareness is regulated by dopamine through the medial prefrontal/anterior cingulate cortex and that this occurs via the GABA system [4]. Dopamine activation in humans elicits GABA activity directly in interneurons in the medial prefrontal/anterior cingulate cortex of the DMN and the associated right insula, thus accounting for the effect of dopamine in its regulation and also for the preferential role of the right hemisphere in self-recognition [70]. This argument is in line with previous research showing how dopamine may have an important mediator role in self-awareness, by increasing gamma power through the medial prefrontal/anterior cingulate cortex [71]. Levels of synaptic GABA are regulated by dopamine release and it is GABA activation that leads to the periodic inhibition of pyramidal cells that results in synchronization of oscillatory activity across brain regions [72]. Attenuation of the normal dopamine regulation of GABA neurotransmission may underlie impaired self-control and self-awareness [54], and imbalances in dopamine and GABA levels or activity could lead to other chronic disturbances in self-awareness, such as in schizophrenia or autism [73].

In addition to the relationship between dopamine and GABA regulating gamma frequency (and, potentially, self-awareness), other neurotransmitters, such as acetylcholine [74], serotonin [75], and oxytocin [76, 77], may interact with GABA interneurons, making those interneurons what Lou et al. [4] call “a natural mini-brain”, thus balancing a wide spectrum of neurotransmitters.

### Dopamine, the SN, and Self-Awareness

Dopamine neurons seem to have an important role when an organism needs to identify behaviourally relevant stimuli [45, 78, 79]. Mesolimbic dopamine neurons (projecting from the ventral tegmental area to the limbic striatum) have been proposed to signal reward prediction errors, indicating the discrepancy in observed and predicted values of a certain stimulus [78, 79]. This association between dopamine neurons and stimuli signalling is one of the main features indicating the role of dopamine in the SN [80]. Supporting this hypothesis, McCutcheon et al. [45] showed that greater limbic dopamine synthesis capacity was directly associated with stronger connectivity within the SN.

Seeley [81] argues that the SN hubs are responsible for the conscious integration of autonomic feedback and responses with internal goals and environmental demands. In this sense, the SN has an important role in maintaining homeostasis, since it can inform the organism what is environmentally relevant based on its current biological needs. Seeley [81] proposes that interactions within the SN could form an information processing loop for representing and responding to homeostatically relevant internal or external stimuli, imbuing them with emotional weight. This hypothesis is also supported by studies that indicate a particular importance of the SN in interoceptive awareness (e.g. [82, 83]).

Alterations in the mechanisms that regulate salience have been linked to several neuropsychiatric conditions. In particular, the aberrant salience hypothesis has been suggested in the context of psychosis [80, 84]. According to this framework, dysregulated dopamine transmission is linked to a stimulus-independent release of dopamine in psychosis [80]. This alters the usual process of contextually driven salience attribution, leading to the aberrant assignment of salience to external or internal stimuli [80, 84]. Several studies have shown that patients with schizophrenia show a heightened synthesis of dopamine [85, 86], heightened dopamine release in response to an impulse [87, 88], and heightened levels of synaptic dopamine [89]. Additionally, antipsychotic medication reduces positive symptoms, such as delusions, acting primarily through the antagonism of dopamine D2/3 receptors [90].

Huang et al. [91] indicate that SN patterns of functional connectivity (FC) may be a transdiagnostic difference between depression and schizophrenia, playing a critical role in the pathogenesis of these two disorders. Their findings suggest that SN-DMN connectivity is significantly associated with both schizophrenia and depression; while FC between these two regions was enhanced in the group with schizophrenia, the group with depression had decreased FC. The authors argue that the decreased FC suggests an anticorrelation, which may reflect an active downregulation or inhibition often found in depression. Altered FC in schizophrenia, unlike in depression, also suggests a disconnection between SN and subcortical caudate [91].

Zhou et al. [92] suggest that alterations in SN may be associated with different patterns of dementia. Early behavioural-variant frontotemporal dementia (bvFTD) disrupts complex social-emotional functions (which can result in impaired social behaviour) by degenerating regions associated with the SN, while posterior cortical regions part of the DMN are initially spared. In contrast, they argue [92] that Alzheimer’s disease often preserves SN connectivity and social-emotional functioning, but is linked to damage to the DMN regions, disrupting memory capacity, including autobiographical memory. This indicates a potential double-dissociation within subtypes of dementia, with different alterations to selfhood linked to SN or DMN lesions.

### The Role of Serotonin on DMN Activity and Other Neural Networks

In a recent review, Conio et al. [93] discuss how the complex interplay between serotonergic and dopaminergic circuits operates to control our attention to objects in the world (increased or decreased salience) and to ourselves (increased or decreased self-reference). They indicate that both dopamine and serotonin nuclei diffusively project to subcortical and cortical regions of resting-state networks (RSNs) [94, 95]. For example, the dopaminergic nigrostriatal pathway is structurally and functionally connected with core regions of the sensorimotor network (SMN), whereas the mesocorticolimbic pathway is connected with core regions of the SN [94, 96]. The serotonergic raphe nuclei (RNi) connections involve regions of the SMN and DMN [94]. Evidence indicates that changes in neurotransmitter activity impact the functional configuration and level of activity of RSNs, as measured by FC and amplitude of low-frequency fluctuations/temporal variability of BOLD signal [97, 98]. Dopamine signalling would be associated with an increase in FC and activity in the SMN (potentially through the nigrostriatal pathway) and SN (via the mesocorticolimbic pathway), as well as a concurrent decrease in FC and activity in the DMN [99]. Serotonin signalling (via the RNi-related pathways), on the other hand, would be associated with a decrease in SMN activity along with an increase in DMN activity [96, 100]. In this perspective, dopamine signalling would favour the predominance of SMN-SN activity, whereas serotonin signalling would favour the predominance of DMN activity, manifesting in distinct behavioural patterns [93].

Additionally, one of the central ideas of their model is that alterations in dopamine and serotonin signalling may result in functional reorganization of RSNs, manifesting in distinct psychopathological states. Three different scenarios are hypothesized [93]. First, deficits in serotonin signalling may lead to DMN deficits with a relative predominance of SMN-SN activity, something that is usually observed in states of mania, in which there is low self-reference and high salience of external objects. Second, deficits in dopamine signalling may result in SMN-SN deficits with a relative predominance of DMN activity, usually seen in depressive states, in which self-reference is high but the salience and emotional value of external objects is low. Third, hyperactive dopamine signalling may be linked to over-activity of SMN-SN, which is usually seen in psychotic states, in which both self-reference and salience of external objects usually are high.

## Self-Awareness and Psychedelics

### A Framework for Understanding the Action of Psychedelics on Self-Awareness

Within the context of self-awareness, the effects of psychedelics may be understood through the lens of a predictive coding (PC) framework, a theoretical model that posits the brain as an inference machine, constantly generating predictions about sensory inputs and minimizing the discrepancies (prediction errors) between predictions and actual sensory data [101]. This framework, initially developed to elucidate external perception [102, 103], has been extended to encompass predictions about internal states (e.g. [104, 105]). The framework has also been considered in relation to self-awareness in general [2, 3] and some of its specific processes (e.g. interoception [5], agency [106]).

PC considers both top-down (predictions influencing perception) and bottom-up (sensory data influencing predictions) processes in neural processing. Top-down generated brain predictions constitute expectations about what sensory information is anticipated to be encountered. These biased predictions are sent from higher brain regions to lower ones, and are influenced by past experiences, learning, and contextual cues. The actual sensory input from the environment is referred to as bottom-up sensory information. When there is a mismatch between the predictions and the actual sensory input, a prediction error signal is generated, which allows either updating predictions or resampling sensory information, thus minimizing future prediction errors. By doing this, the brain creates a representation of the world, which needs to be sufficiently functional from an evolutionary perspective, i.e. verisimilar enough to lead to survival.

Psychedelics are hypothesized to alter perception processes by affecting the precision of high-level priors or beliefs, thereby facilitating bottom-up information flow through a high Bayesian learning rate [107]. By dampening the filtering mechanisms that normally prioritize familiar sensory information, psychedelics would allow a broader range of sensations and emotions to reach subjective awareness. This can lead to heightened sensitivity to subtle physiological cues, emotions, and bodily sensations that are normally overlooked or suppressed by typical neural filtering mechanisms.

Modulations by psychedelics in cerebral blood flow and neural connectivity, especially on important self-related networks, such as the DMN and SN, would enable a broader range of alterations in self-awareness (e.g. [25, 108, 109]. Moreover, psychedelics seem to lead to increased entropy of the degree distribution (i.e., network heterogeneity) for the functional brain networks (e.g. [110–112]), generating a wide range of changes in cognition and altered states of consciousness. Within this perspective, the Relaxed Beliefs Under Psychedelics (REBUS [113]) model has suggested that psychedelics, through entropy changes and self-related network disruptions, have the potential to ease the precise weighting of existing pre-concepts and beliefs, simultaneously enhancing the flow of information from bottom-up processing.

An alternative, but somewhat similar, explanation refers to the evidence pointing out that the impact of psychedelics in self-related processes might arise due to disruptions in gating mechanisms, stemming from a breakdown in the processing of information within feedback loops connecting the cortico-striatal-thalamo-cortical pathway [114], as well as due to disruption in higher cortical networks through cortico-claustral-cortical circuitry [115]. In any case, these competing models have limitations in accommodating current data and need more supporting evidence from empirical studies [116].

### Impact of Psychedelics on Specific Self-Awareness Processes

As indicated previously, self-awareness can be understood as a variety of processes. A non-exhaustive taxonomy has been suggested [2, 3], including processes such as interoception (sensory information about visceral states), proprioception/body ownership (the feeling of owning a body and identifying its positioning in space), agency (the sense of generating one’s actions), metacognition (monitoring and regulation of cognition), emotional regulation (monitoring and regulation of emotion), and autobiographical memory (records of personal information and incident memory). These processes have been highlighted for a variety of reasons. Firstly, they can be observed across cognitive complexity levels, from basic sensory information to higher-order reflexive abilities, within humans and even in other species [2, 117]. At lower complexity levels, some of these abilities may operate through implicit processes, with recursive higher-order awareness being exhibited only for more complex phenomena. Secondly, this taxonomy considers that self-awareness has two core dimensions: bodily self-awareness (represented by interoception, body ownership/proprioception, and agency) and self-representations (metacognition, emotional regulation, and autobiographical memory). These sources of self-awareness interact with each other, leading to an emergent unified sense of self (for a more detailed description, see Mograbi et al. [3]). Finally, these processes correspond to fundamental features for sustaining organic entities, such as self-monitoring (e.g. interoception, metacognition), internal/external boundaries (e.g. body ownership), action in the environment (e.g. agency), regulation (e.g. emotional regulation, metacognition) and registering of information (e.g. autobiographical memory). This suggests that self-awareness is an adaptive feature and not merely an evolutionary epiphenomenon or by-product [117].

The impact of psychedelics on these self-awareness processes is discussed below and summarised in Table 1. It is important to emphasize that the subjective effects of psychedelics can vary significantly depending on many factors, such as the substance, personal characteristics of users (e.g. personality, mood), setting aspects, and crucially, the dosage used. The range of alterations of self-awareness is therefore potentially modulated by many variables. For instance, it seems to be consensual that the sensation of ego dissolution is dose-dependent and that high doses of psychedelics can profoundly alter the sense of having a preserved self [118, 119]. Furthermore, it is important to consider the influence on self-awareness concerning the acute, sub-acute, and chronic effects of psychedelics. Additionally, there are few studies that have aimed to investigate alterations occasioned by psychedelics in self-awareness processes through specific experimental tasks for each process.

**Table 1:** Effects of psychedelics on self-awareness

Self-awareness processes	Definition	Clinical conditions/symptoms
Interoception	Sense of the physiological conditions of the entire body	Increases with low but not microdoses of classic psychedelics. Increased interoceptive change in depression with ayahuasca. Increases with low and medium, but impairment with high doses of salvinorin-a.
Proprioception and body ownership	Mapping of the relative position of body parts, and the feeling of owning a body, respectively	Sense of disembodiment in acute psychedelic experiences, with potential stronger emotional embodiment later on. Loss of body ownership with high-doses of salvinorin-a, but increases in bodily sensations with low and medium doses. Disruption of body ownership with ketamine.
Agency	Sense of generating our own actions	Potential increases after classic psychedelic microdosing, impairments after ketamine use.
Metacognition	Monitoring, knowledge and regulation of cognition	Metacognitive deficits in regular MDMA users and after acute ketamine use.
Emotional regulation	Monitoring and regulation of emotion	Short- and long-term benefits of classic psychedelics. Negative effects of ketamine in healthy users, but positive outcomes in depression. Positive effects of MDMA in PTSD.
Autobiographical memory	Records of self-information, including specific episodes and general knowledge about oneself	Classic psychedelics reduce mental time travel and intensify vividness of memories. Enhancement of emotionally positive memories by MDMA-assisted therapy

#### Interoception

Interoception may be a useful example to examine the relationship between self-awareness and PC. It is hypothesized that mismatches between interoceptive signals and inferences may explain certain pathological changes in self-awareness [120]. There are a few reports suggesting that classical psychedelics cause subjective interoceptive feelings, although these effects have not been fully specified. For instance, in a study by Carr & Lucki [100], a low dose of ayahuasca produced high interoceptive and somatic effects reported by users in comparison to placebo. By contrast, a study by Marschall et al. [121] revealed that microdoses of psilocybin did not affect self-reported interoceptive awareness. The microdoses used in this study were composed by 0.7 g of dried psilocybin-containing Galindoi truffles, which corresponds to around 1/10th of a medium-high dose.

Using quantitative textual analysis, Cruz et al. [122] have shown how one of the central axes of the experience reported by first-time ayahuasca users refers to somatic changes, such as nausea, hunger and breathing changes. Notably this effect seems to be more pronounced for people with depression in comparison with healthy controls. Despite these findings, there is a lack of studies objectively measuring interoception in relation to classic psychedelics.

A placebo-controled study by Maqueda et al. [123] reported interoceptive changes caused by salvinorin-a - the main psychoactive molecule found in Salvia divinorum, which, despite not being a classical psychedelic, can produce psychedelic-like states [124]. In this study, low and medium doses of the substance led to an increase in experiencing one’s body as safe and trustworthy, while high doses produced significant impairment in interoceptive awareness [123].

#### Body Ownership

A sense of disembodiment is widely reported in the acute psychedelic experiences [125]. For instance, “dread of ego dissolution” is a dimension of some of the most used self-report measures to investigate subjective effects of psychedelic experience (e.g., Dittrich’s Abnormal Mental States, APZ [126]; its shortened and revised versions, OAV, [127]; 5D-ASC [128, 129]). In this situation, users commonly report anxiety, a sense of disembodiment and/or other bodily-/self-alterations. In a study by Preller et al. [114], feelings of disembodiment and other subjective effects after oral consumption of 100μg of LSD correlated with significant changes in connectivity in the SMN. The feeling of body detachment in psychedelic acute experience is hypothesized to be related to losses in temporal perception [130].

Acutely, psilocybin seems to produce aberrant prediction error processing of tactile mismatch responses that generate subjective alterations in bodily self-awareness [131]. However, the specific mechanisms and duration of these changes remain unclear. In a study on psilocybin-assisted therapy for treatment-resistant depression conducted by Watts et al. [132], participants’ reports revealed the perception of specific bodily changes associated with the relief of depressive symptoms, including feelings of emotional embodiment and stronger bodily connection after treatment.

Regarding non-classical psychedelics, there is evidence that low and medium doses of salvinorin-a lead to an increase in bodily sensations, while high doses lead to profound loss of body ownership and out-of-body experiences [123]. It was also observed by Morgan et al. [133] that healthy participants displayed a notable elevation in susceptibility to a distorted perception of limb ownership measured through the moving rubber hand illusion upon ketamine exposure. The effects of ketamine resembled the disrupted sense of body ownership observed in schizophrenia not only acutely, but also after chronic exposure [134], indicating that ketamine may induce a similar modification seen in schizophrenia in the integration of information across sensory domains.

#### Sense of Agency (SoA)

A study by Polito & Stevenson [135] explored responses to the Sense of Agency Rating Scale (SOARS [136]) in a sample of psychedelic microdose users. SOARS is a self-report scale that measures SoA through actions and life experiences, being associated with hypnotisability. They found an increase in reported SoA on dosing days but without long-lasting changes. It can be argued, however, that the construct measured by SOARS is closer to sense of purpose than agency. Beyond classic psychedelics, ketamine may acutely alter SoA, changing perception of the timing of a causal action and mimicking the performance of people with schizophrenia in intentional binding tasks [137, 138].

The notion of SoA over thoughts has been hypothesized in neuroscience, but it is still debatable [139]. The concept refers to the perception of oneself as the agent of mental actions, which would be compromised in individuals experiencing specific psychotic symptoms [140]. It is argued that SoA may be elicited by metacognitive representations of thinking processes that are used for intentional inhibition of thoughts [139]. It can be argued that psychedelics may temporarily reduce SoA over thoughts, generating a dreamlike state composed of thoughts and imagery that emerges through consciousness without a perceived intention or inhibition of the experiencer. However, there is evidence (e.g. [141, 142]) suggesting that psychedelics elicit visual hallucinations principally through the activation of prefrontal cortical or temporal areas, as well as top-down spreading of activation to parietal and primary visual regions, mirroring the mechanism underlying normal visual mental imagery [143]. This contrasts with the hypothesis that psychedelic-induced vivid mental imagery is caused by bottom-up driven neuronal activity.

#### Metacognition

Despite a scarcity of studies directly exploring the impact of psychedelics on metacognitive processes, it has been hypothesized that psychedelic experiences are associated with improvements in metacognitive awareness and consciousness clarity [143]. There is no research directly investigating metacognition and classic psychedelics, but two studies explored the impact of MDMA and ketamine. There is evidence of metacognitive deficits in relation to subjective and objective memory in regular MDMA users, in addition to significant memory impairment in relation to non-users [144]. Considering that a direct relationship between metacognition for memory ability (metamemory) and memory has been shown in some cases (e.g. [145]), it is not clear whether the metacognitive impairment observed in MDMA users is primary or secondary in nature. Ketamine seems to acutely induce deterioration in metacognitive performance in a perceptual decision-making framework [146] and may have deleterious effects on metacognitive sensitivity, leading to larger metacognitive biases during a memory retrieval task [147].

#### Emotional Regulation

It is well known that psychedelics can profoundly alter emotional and affective states, but the impact of psychedelics on emotion regulation processes still needs to be fully understood. It has been noted that psychedelics decrease amygdala reactivity to negative stimuli (but see Effinger et al. [148]), acutely diminishing negative emotional and cognitive biases that are features of depression [149], and this is associated with increases in positive mood [150]. However, in a study with psilocybin [116], the reduced amygdala response to negative stimuli, as well as the decrease in negative affect, returned to baseline one month after administration.

A study by Kometer et al. [151] with healthy individuals provided evidence that psilocybin acutely shifts emotional biases and that promotion of 5-HT2A receptor activation plays a central role in regulating mood. A recent prospective longitudinal study [152] revealed that naturalistic psilocybin use is associated with enduring improvements in emotion regulation abilities. In a study investigating the impact of psychedelic use on domestic violence perpetration [153], men who endorsed a lifetime history use of LSD or psilocybin mushrooms reported better emotion regulation when compared to males with no history of psychedelic use. However, considering the observational design, a causal relationship cannot be established.

A study by Domínguez-Clavé et al. [154] revealed that ayahuasca increases emotion regulation and mindfulness abilities in the 24-hour period after the intake, especially in individuals with borderline-like traits, who suffer significantly with emotional dysregulation. In addition, there is evidence [155] that ayahuasca helps to improve self-compassion and self-criticism capacities in the 24-hour period after the intake, which could have therapeutic implications on emotional regulation. Moreover, a recent fMRI study by Sanchez et al. [156] revealed an acute improvement in emotion regulation mechanisms promoted by ayahuasca in experienced users in response to aversive stimuli. The study by Agin-Liebes et al. [157] suggests that therapeutic outcomes by ayahuasca ceremonial use is due in part to improvements in emotion regulation processes and cognitive reappraisal.

A meta-analysis conducted by Galvão-Coelho et al. [13] revealed a moderate effect size for positive mood impact of psychedelics in a longer-term analysis (between 16 and 60 days after interventions). The impact of psychedelics on emotional regulation processes may be key to comprehending the duration of positive effects of classical psychedelics on mood.

Regarding non-classical psychedelics, ketamine seems to acutely impair emotion regulation, generating emotional bluntness [158] and, during emotional memory formation, increases in amygdala and orbitofrontal reactivity for negative stimuli, leading to general and specific emotion effects that overlap with alterations observed in schizophrenia [159]. Conversely, there are data on ketamine enhancing neural responses specifically to positive emotions in patients with treatment-resistant depression [160]. In an uncontrolled trial conducted by Monson et al. [161], MDMA was combined with Cognitive-Behavioural Conjoint Therapy (CBCT) for post-traumatic stress disorder (PTSD) and produced significant improvements in depressive symptoms and emotion regulation abilities.

#### Autobiographical Memory

There is substantial evidence that classical psychedelics acutely modulate autobiographical memory. It is hypothesised that these alterations are driven specifically by alterations in mental time travel – the human ability to mentally project backward and forward in time, recalling past autobiographical episodes or imagining future experiences [162]. A review conducted by Healy [163] showed that experimental research indicates that classical psychedelics, such as LSD and psilocybin, seem to reduce the typical mental time travel to the past and intensify the vividness, visual characteristics, and childlike/primitive qualities of autobiographical memories. Qualitative investigations and clinical case reports further suggest that psychedelics may entail the recollection and reliving of emotionally charged autobiographical memories, some of which were previously avoided or forgotten [163–165]. There is evidence that decreased mental time travel after LSD consumption is significantly correlated with DMN disintegration [166]. Moreover, there is evidence that the reported enhancement of vividness and visual qualities in autobiographical recollections under psychedelics is also associated with a heightened neural activity in visual and other sensory regions of the brain (e.g. [167]).

Furthermore, the therapeutic potential of psychedelics in the context of autobiographical memory is being explored. MDMA-assisted therapy, in particular, has shown promise in the treatment of PTSD. In a study by Carhart-Harris et al. [112], autobiographical memories of participants who took MDMA appeared more vivid, emotionally intense, and positive in comparison to a placebo group, and negative memories were rated as less aversive, indicating an enhancement of emotionally positive memories by MDMA. As indicated, this may be directly linked to the therapeutic potential attributed to MDMA. For instance, the degree of PTSD symptoms reduction following MDMA-assisted therapy in a study by Singleton et al. [168] was associated with alterations in four functional connections during the recall of autobiographical memories, including the left amygdala to left PCC, left amygdala to right PCC, left amygdala to left insula, and left isthmus cingulate to left posterior hippocampus.

## Summary of Findings, Potential Mechanisms, and Future Research Directions

Although existing evidence is still scarce, some patterns emerge from the reviewed results. In particular, classic psychedelics, such as ayahuasca, LSD, and psilocybin, seem to provide both short- and long-term benefits for emotional regulation. Improved outcomes can be seen not only in regulation itself but also in the modulation of autobiographical memory, for example with improved capacity to relive emotional memories (for classic psychedelics) or positive enhancement of memory (in the case of MDMA).

This improvement in emotional regulation is consistent with a recently suggested approach to explain the action of classic psychedelics, namely Carhart-Harris & Nutt's [169] ‘bipartite’ model of brain serotonin function. This model proposes that brain serotonin mediates adaptive responses to adversity via two distinct mechanisms: one mediated by postsynaptic 5-HT1AR signalling assisting with stress moderation and the other mediated by 5-HT2AR signalling linked to more substantial adaptive changes. Postsynaptic 5-HT1AR is thought to be the principal (therapeutic) site of action of SSRIs [170, 171], by promoting stress tolerance and relief (i.e. passive coping). On the other hand, cognitive flexibility in humans is thought to be positively modulated by 5-HT2AR functioning [172] and there is evidence to suggest that 5-HT2AR agonists (such as psilocybin and LSD) enhance cognitive flexibility and creative thinking [173–176], potentially in an enduring way [174]. By providing flexibility to higher-order priors and beliefs, psychedelics would allow different interpretations of incoming sensory information, expanding the repertoire of potential answers to internal and external stimuli. Carhart-Harris & Nutt [169] also hypothesize that active coping can be most effectively implemented if the window of plasticity afforded by 5-HT2AR agonism is complemented by supportive psychotherapy that promotes a willingness to confront and work through sources of stress [132].

The findings on emotional regulation and autobiographical memory suggest that psychedelics may have a particular impact on narrative features of self-awareness. The exception would be metacognition, for which there is no evidence for the effects of classic psychedelics, but results pointing to negative effects of ketamine (acute) or MDMA (chronic) use. Further research is needed to clarify how metacognition can be modulated by psychedelics, not only in the context of direct investigations of this process but also in clinical studies with conditions in which metacognition is a prominent feature, such as obsessive-compulsive disorder [177]. It is possible that clearer effects on emotional regulation and autobiographical memory have been observed because of larger emotional components in these processes in relation to metacognition, so future research may want to investigate, in the context of psychedelic use, features of metacognition with varied levels of emotional engagement. For instance, electrophysiological investigations of error-related event-related potentials, such as error-related negativity, positivity, and feedback-related negativity, have manipulated the emotional load of errors (e.g. [178]). Such a study, in the context of psychedelics, may shed light on the mechanisms of these substances in affecting awareness of performance, errors, and cognitive ability.

Findings on bodily self-awareness are scarcer and less consistent. Two key factors that warrant further empirical exploration are the doses employed and the time course of changes. Comparing the threshold at which specific molecules exert physiological, behavioural, or subjective effects is a relevant research direction; for instance, in the case of interoception, while microdoses of psilocybin caused no observable changes, low doses of ayahuasca led to reported effects. It is possible that the impact of psychedelics on bodily self-awareness may not follow a monotonic dose-response curve, as indicated by findings with salvinorin-a, suggesting positive effects with low and medium, but impairments with high doses [123]. Future studies should investigate how micro, low, medium and high doses affect self-awareness processes, exploring the dose-response curves for different substances.

In relation to the time course of changes to self-awareness, research into embodiment indicates impairments during the experience with substances such as psilocybin and LSD [179], but it is possible that this leads to enhanced embodiment later on, which may account for therapeutic effects [132]. More longitudinal investigations, teasing apart the short- and long-term effects of psychedelics on self-awareness are needed. One prediction, based on the REBUS model, is that rearrangement of priors after the psychedelic experience may lead to sustained positive outcomes. Future studies may explore empirically if self-awareness changes during the acute phase are predictors of clinical response.

The presence of psychopathological conditions may also be an important mediator of the changes in self-awareness driven by psychedelics. Ketamine, for instance, is typically linked to worse outcomes in terms of self-awareness variables, and, yet, it has shown consistent results as a quick-acting antidepressant [180]. Given how self-awareness variables can be negatively affected by depression (e.g. interoception [181] and metacognition [182]), it is fair to assume that ketamine may induce positive changes in these processes in people with depression, primarily or through secondary mechanisms. Findings by Cruz et al. [122] indicated a stronger interoceptive component in the experience of first-time ayahuasca users with depression relative to healthy controls, but the scarcity of studies directly comparing the effects of psychedelics on self-awareness of people with and without clinical conditions highlights this as an important future research direction.

An avenue of research that has been given less attention in recent years is the use of psychedelics to model psychopathology phenomena. The effects of psychedelics on body ownership and SoA may be particularly relevant for this purpose. Findings from Tang et al. [134] do seem to indicate body ownership disruptions from ketamine may be similar to those in schizophrenia. Given that SoA is typically affected in psychosis, investigating which substances can lead to changes in this ability may help in developing chemical models for this condition. This may clarify to which extent psychedelics and related substances can really be used as psychotomimetic agents.

Such investigations may benefit from the frameworks described by Lou et al. [4] and Conio et al. [93], indicating that the modulation of DMN and SN by neurotransmitters such as GABA, dopamine, and serotonin may be behind a number of psychopathological phenomena linked to self-awareness. Understanding psychopathology symptoms through the prism of these RSN networks and the effects that different molecules may have in rebalancing connectivity between them may lead to novel therapeutic approaches and the refinement of existing treatments. One prediction is that classic psychedelics act mainly through changes in DMN, which would explain the therapeutic effects observed particularly in conditions with excessive DMN functioning (e.g. psilocybin for depression, [14]), and is also in line with more consistent changes in narrative features of self-awareness, as described here. However, that may be a fairly simplistic approach, given it does not account for the therapeutic promise in externalizing conditions (e.g. LSD or ayahuasca for addiction, [183, 184]), in which SN is heavily involved and dopamine alterations are a prominent feature. A more comprehensive approach is needed, in which substances are considered in terms of how their binding affinities may directly affect self-related networks, but also considering the dynamic relationship between RSN. Investigating the interplay between RSN across clinical conditions and substance use effects may provide the resources for such an approach, clarifying the relationship between psychopathology and self-awareness, and paving the way for the development and refinement of pharmacological interventions and psychedelic-assisted psychotherapy (PAP).

Finally, exploring the relationship between self-awareness and consciousness in general may indicate mechanisms, neural correlates and neurochemical influences shared by these processes. Self-awareness contributes with essential information for consciousness, mapping internal states and capacities, but it is also reliant on the neural structures that support conscious activity, such as the reticular formation and intralaminar nuclei. Future research should explore the extent to which alterations in consciousness in general impact on self-awareness. Additionally, how and why specific processes remain implicit, while others emerge to consciousness, remains an unanswered question. A neurochemical approach for both these questions may provide important advancements for mechanistic explanations about consciousness and self-awareness, while also assisting in the management of clinical conditions characterised by impairments of consciousness.

## Conclusions

There are several limitations in the provisional framework to understand the action of psychedelics on self-awareness presented here. Despite considerable progress in the past decades, current knowledge of the neurochemical basis of cognition is still limited. The study of psychopathological alterations has been a pathway for a better understanding of how neurotransmitters are implicated in mental phenomena. Nevertheless, with few exceptions (e.g. Parkinson’s disease), the neurochemical hypotheses for different disorders are insufficient to explain clinical presentation and treatment response. Even in cases where progress has been made, such as in the case of the dopaminergic hypothesis of psychosis, limited features of the condition are explained – delusions can be more easily accommodated to the aberrant salience hypothesis than hallucinations or negative symptoms [185]. Competing and/or complementary hypotheses (e.g. the role of glutamatergic and serotoninergic pathways in psychosis [186]) may provide a more nuanced description of mechanisms, but not necessarily translate into better treatments (e.g. the abandonment of pomaglumetad as a potential new intervention for psychosis [187]).

Difficulties in translating knowledge about neurotransmitters into clinical practice also demonstrates the limitations of current diagnostic approaches. As long as patients are understood as belonging to discrete categorical descriptions, as opposed to diverse phenotypes exhibiting phenomena that may cross diagnostic boundaries, progress is bound to be limited. Future studies on self-awareness may benefit from a focus on the relationship with specific symptoms, in a transdiagnostic approach.

A research program investigating how substances can impact different self-awareness processes may bring direct benefits not only for the development of new treatments but also for our understanding of the subjective experiences of clinical groups. This approach would benefit from experimental investigations exploring specific self-awareness processes in a structured way, comparing different dosages and substances, but also from clinical studies. For example, investigating autobiographical memory in the context of PAP may illuminate the mechanisms through which change and clinical improvement are promoted and how this is perceived by patients.

Although most psychedelics continue to be illegal worldwide [188], growing efforts have been made to legalize and regulate the clinical use of some of these substances [189]. Australia has already authorized the prescription of MDMA and psilocybin under professional supervision in 2023 [190]. In the United States, the Federal Drug Agency (FDA) announced its first draft guidance for psilocybin in 2023, suggesting that it will approve further clinical trials that investigate its therapeutic potential [191], and considers MDMA-assisted interventions for PTSD, with clinical trials currently at the phase 3 stage and close to obtaining regulatory approval, a “breakthrough therapy” [192]. Esketamine, an enantiomer of ketamine, has been approved for treatment-resistant depression by the FDA and other countries (e.g. Brazil), and can now be prescribed by medical professionals in these regions. These regulatory changes highlight the promise of psychedelic-based interventions, suggesting that researchers and clinicians alike should pay attention to the advancement of this field.

The use of psychedelics has a long history, with rich descriptions of their effects from a subjective point of view. Bridging the gap between the phenomenology of altered states of consciousness, including in the case of clinical treatments, and physiological descriptions is one of the current frontiers of knowledge. This will be more easily achieved if future studies use complementary methods, privileging both self-reported experiences and the measurement of brain states and behavioural responses. This is in line with the potential paradigm shift brought by PAP, in which instead of relying on a primarily pharmacological model, focus is given to the synergy between contextual, pharmacological and psychological aspects of the patient [193]. In this perspective, the pharmacological intervention occurs sporadically, in the presence of therapists (usually two), who will help defining expectations, managing and, posteriorly, integrating the experience [194]. An integrative approach, both for research and clinical work, may pave the way for a fuller understanding of and capacity to deal with the human condition, including of some of our long-lasting questions, such as self-awareness.

## Key References

• Mograbi DC, Huntley J, Critchley H. Self-awareness in dementia: A taxonomy of processes, overview of findings, and integrative framework. Curr Neurol Neurosci Rep. 2021;21(12):69. https://doi.org/10.1007/s11910-021-01155-6.

- This article introduces the taxonomy of self-awareness used here, discussing a framework to understand this process.

• Mograbi DC, Hall S, Arantes B, Huntley J. The cognitive neuroscience of self-awareness: Current framework, clinical implications, and future research directions. Wiley Interdiscipl Rev: Cogn Sci. 2023:e1670. https://doi.org/10.1002/wcs.1670.

- This article presents a PC framework to understand self-awareness, describing the potential relationship between different processes to generate an emergent sense of self-awareness.

• Lou HC, Changeux JP, Rosenstand A. Towards a cognitive neuroscience of self-awareness. Neurosci & Biobehav Rev. 2017;83:765–73. https://doi.org/10.1016/j.neubiorev.2016.04.004.

- Seminal work on the field, identifying potential relationships between neurotransmitters and self-awareness.

• Snyder AZ, Raichle ME. A brief history of the resting state: the Washington University perspective. Neuroimage. 2012;62(2):902–10. https://doi.org/10.1016/j.neuroimage.2012.01.044.

- An overview of the historical basis of the concept of DMN, by researchers directly involved in its research.

• Seeley WW. The salience network: a neural system for perceiving and responding to homeostatic demands. J Neurosci. 2019;39(50):9878–82. https://doi.org/10.1523/JNEUROSCI.1138-17.2019.

- A description of the functioning of the SN by one of the main researchers involved in its conceptualization.

• Huang H, Chen C, Rong B, Wan Q, Chen J, Liu Z, et al. Resting-state functional connectivity of salience network in schizophrenia and depression. Sci Rep. 2022;12(1):11204. https://doi.org/10.1038/s41598-022-15489-9.

- Empirical work showing different patterns of SN connectivity in psychosis and depression.

• Conio B, Martino M, Magioncalda P, Escelsior A, Inglese M, Amore M, Northoff G. Opposite effects of dopamine and serotonin on resting-state networks: review and implications for psychiatric disorders. Mol Psychiatr. 2020;25(1):82–93. https://doi.org/10.1038/s41380-019-0406-4.

- A thorough review on how dopamine and serotonin impact SN and DMN activity generating different profiles of psychopathology.

• Carhart-Harris RL, Friston KJ. REBUS and the anarchic brain: toward a unified model of the brain action of psychedelics. Pharmacol Rev. 2019;71(3):316–44. https://doi.org/10.1124/pr.118.017160.

- The authors suggest that psychedelics work by relaxing the precision of high-level priors, a framework that can be applied to self-awareness.

• Carhart-Harris RL, Nutt DJ. Serotonin and brain function: a tale of two receptors. J Psychopharmacol. 2017;31(9):1091–120. https://doi.org/10.1177/0269881117725915.

- A framework for understanding the differential action of SSRI and psychedelics, based on the binding affinities of these substances, is presented.

## Data Availability

No datasets were generated or analysed during the current study.
